# Primary and Posttraumatic Arthritis of the Elbow

**DOI:** 10.1155/2013/473259

**Published:** 2013-05-27

**Authors:** Debdut Biswas, Robert W. Wysocki, Mark S. Cohen

**Affiliations:** Section of Hand and Elbow Surgery, Department of Orthopaedic Surgery, Rush University Medical Center, 1611 W. Harrison Street, Chicago, IL 60612, USA

## Abstract

Whether degenerative joint disease of the elbow may be the result of primary or posttraumatic etiologies, arthritis of the elbow commonly leads to pain, loss of motion, and functional disability. A detailed history and focused physical examination, in combination with imaging modalities, can help localize the origin of symptoms and help direct treatment. Although nonoperative treatment is the initial therapy for arthritis of the elbow, surgical interventions may provide substantial relief to the appropriately selected patient.

## 1. Introduction

Degenerative joint disease of the elbow may be a painful condition for a majority of patients. Although primary osteoarthritis is less common than posttraumatic arthritis of the elbow, both conditions result in symptoms which affect the quality of life. The health care provider encountering these conditions must carefully tailor treatments, both nonoperative and operative, to account for the patient's age, personal preferences, functional demands, and severity of arthritic changes. 

This paper will review the pathogenesis of both primary and posttraumatic arthritis of the elbow. The principles of nonoperative management will be presented as well as the indications and considerations for operative treatment.

## 2. Background/Pathogenesis

### 2.1. Primary Osteoarthritis

Primary osteoarthritis of the elbow is an uncommon condition which occurs predominantly within the ulnohumeral joint of the dominant extremity of patients who engage in heavy sport or labor. The intrinsic congruity of the ulnohumeral articulation preserves a majority of the articular cartilage, with degenerative changes and osteophytes initially affecting the tips of the olecranon and coronoid processes as well as their respective fossae within the distal humerus. Accordingly, the most common complaint of patients in our practice with early stages of primary osteoarthritis of the elbow is pain at terminal flexion and extension, as the osteophytes of the coronoid and olecranon engage their fossae. As the degenerative process involves more of the articular surface, pain is encountered throughout the arc of motion, and enlarging osteophytes coupled with anterior and posterior capsular contracture may provide further mechanical impediments to motion and result in measurable loss of motion in terminal flexion and extension.

**Figure 1:**
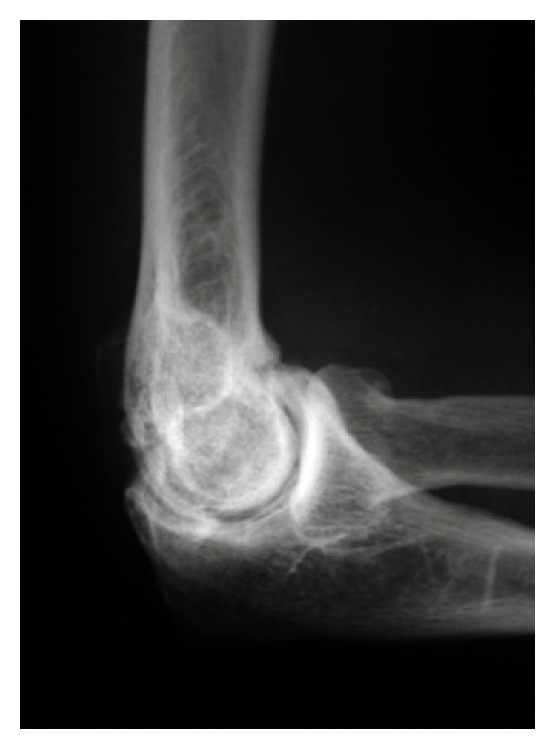
Lateral radiograph demonstrates idiopathic arthritis with osteophytes on the radial head as well as anterior and posterior ulnohumeral joints.

The majority of articular surface involvement is confined to the ulnohumeral joint. Isolated primary osteoarthritis is uncommon within the radiocapitellar articulation. Accordingly, the practitioner should carry out a detailed history and meticulous physical examination in order to localize the origin of symptoms in the patient with primary osteoarthritis of the elbow. Ulnohumeral arthrosis typically results in pain and diminished range of motion in flexion and extension, while radiocapitellar arthrosis predominantly manifests as focal lateral-sided pain over the radiocapitellar joint during forearm rotation (Figure 1).

### 2.2. Posttraumatic Arthritis

**Figure 2:**
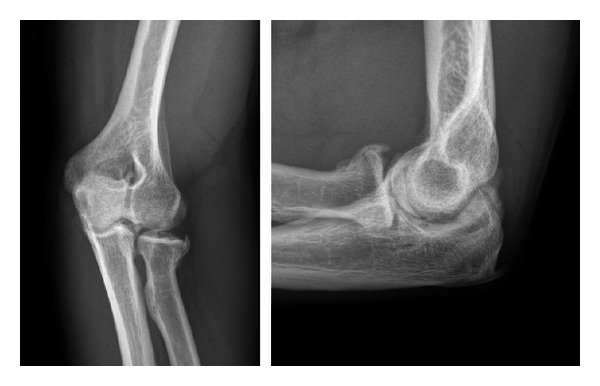
Oblique and lateral radiographs of a patient who sustained a radial head fracture treated nonoperatively; this patient later developed lateral elbow pain with forearm rotation. Radiographs demonstrate anterolateral osteophytes noted at the radial head and decreased joint space in the radiocapitellar joint.

A variety of traumatic insults may ultimately result in specific forms of posttraumatic arthritis to the elbow joint. Radiocapitellar incongruity may result following malunion of an intraarticular radial head fracture (Figure 2); patients will typically report lateral-sided pain and crepitus exacerbated during rotation of the forearm rather than flexion and extension. Conversely, malunion of distal humerus or olecranon fractures which extend intra-articularly into the ulnohumeral joint typically result in arthritis which is symptomatic during flexion and extension.

Although malignments in the geometry of the articular surface alter contact pressures and expedite the development of arthritis, ligamentous instability about the elbow may also result in abnormal joint kinematics and the potential for degenerative changes. These potential injuries should be carefully evaluated, as the presence of elbow instability dramatically alters the treatment objectives in the setting of elbow arthritis, particularly when surgical intervention is considered. 

## 3. Evaluation

### 3.1. History

The most common complaints of patients with either primary or posttraumatic arthritis of the elbow are pain and/or loss of motion; it is important for the practitioner to precisely characterize the principal complaint. Complaints of pain should be localized when possible, particularly when attempting to discern between symptomatic arthrosis of the radiocapitellar or ulnohumeral joint. Pain may be associated at the extremes of motion in earlier forms of ulnohumeral arthritis but may be experienced throughout the arc of elbow flexion and extension in more advanced disease [1, 2]. Although loss of extension is most common, loss of motion may manifest itself as a loss in elbow flexion or forearm rotation depending on the site of arthritis, and it is important to determine if there is true functional disability from the condition, or if the symptoms represent simply a subtle asymmetry when compared to the contralateral side.

For patients who report a prior episode of significant elbow trauma, the details surrounding the initial injury as well as mechanism of trauma are important aspects of the history, as any previous treatments including operative reports from previous surgeries and therapy reports to elicit what treatments have been undertaken. Ulnar neuropathy is a frequent finding in patients presenting with degenerative joint disease of the elbow and is frequently subtle and not part of the patient's chief complaint. The examiner should specifically inquire about sensations of numbness and tingling in the ring and small finger, loss of dexterity, and soreness over the ulnar nerve in the posteromedial elbow, in order to comprehensively evaluate for cubital tunnel syndrome. Although uncommon, any suggestion or suspicion of infection in the setting of previous open trauma, surgery, or septic arthritis requires a thorough evaluation, including serologic markers and aspiration of the joint to be analyzed for synovial fluid cell count, culture, and gram stain.

Finally, the demand level of the patient must be carefully considered, as certain patients may desire nearly full motion for specific lifestyle and employment demands, which would lower the threshold for motion-preserving debridement, while others may require heavy lifting which would preclude treatment with a total elbow arthroplasty. It should be noted that loss of flexion tends to be much more functionally limiting than loss of extension, as the latter can be compensated for simply by moving closer to an object to reach it. A loss of flexion, however, cannot be compensated for. A loss of pronation can be accommodated for by abducting the shoulder, whereas a loss of supination cannot be accommodated for. The willingness of the patient to comply with the required extensive programs of therapy must also be considered, as treatment outcomes depend on diligent participation in a structured rehabilitation program. 

### 3.2. Physical Examination

Physical examination begins with inspection of the entire upper extremity, specifically evaluating for deformity, swelling, and muscle atrophy, while noting the location of any previous surgical incisions that would influence further surgical planning. Range-of-motion evaluation should include the hand, wrist, forearm, and elbow and should be compared with the contralateral, unaffected extremity. In the posttraumatic setting, loss of extension is more common than loss of flexion, whereas in osteoarthritis the loss in motion is quite variable depending on the location of impinging osteophytes.

A careful neurovascular examination is important, especially during the evaluation of ulnar nerve function. Residing in the cubital tunnel and adjacent to the medial joint capsule, the ulnar nerve may become compressed either from scar tissue along the medial elbow following trauma or from thickened capsule, osteophytes, or synovitis. Traction ulnar neuritis of the elbow may manifest as medial elbow pain and patients may complain of sensory changes in an ulnar nerve distribution, particularly with elbow flexion. Patients with posttraumatic ulnar neuropathy may present simply with loss of flexion and medial elbow pain in the absence of overt symptoms of ulnar neuropathy; thus, a meticulous neurovascular evaluation, including two-point discrimination, pinch strength, and intrinsic muscle function are essential to document the preoperative function of the ulnar nerve. Electrodiagnostic testing may be considered in the setting of provocative symptoms of ulnar neuropathy.

Evaluation of elbow stability is essential in order to exclude ligamentous insufficiency as a potential diagnosis. Varus and valgus stresses are applied to the affected elbow with the contralateral elbow serving as a normal comparison. Posterolateral rotatory stability is evaluated with the lateral pivot-shift maneuver; this test is performed by applying a valgus stress and axial load to the partially flexed elbow with the forearm fully supinated. Posterior subluxation of the radial head is pathogonomic for posterolateral rotatory instability; alternatively, pain without subluxation is suggestive of instability [3].

### 3.3. Imaging

Plain radiographs of the elbow are typically obtained and include anteroposterior, lateral, and oblique projections; diligent efforts should be made to review previous imaging for patients presenting with posttraumatic arthritis. Stress radiographs should be considered if elbow instability is suspected. Radiographs typically demonstrate preservation of joint space centrally with osteophytes along the anterior and posterior aspects of the joint which may cause impingement (Figure 1). Radiographs should be carefully examined in the posttraumatic elbow, specifically evaluating for malalignment, incongruency of the ulnohumeral or radiocapitellar joints, or the development of heterotopic ossification (HO). Rettig et al. have suggested that surgical debridement may be less effective for more advanced arthrosis of the elbow joint as visualized on diagnostic imaging [4].

**Figure 3:**
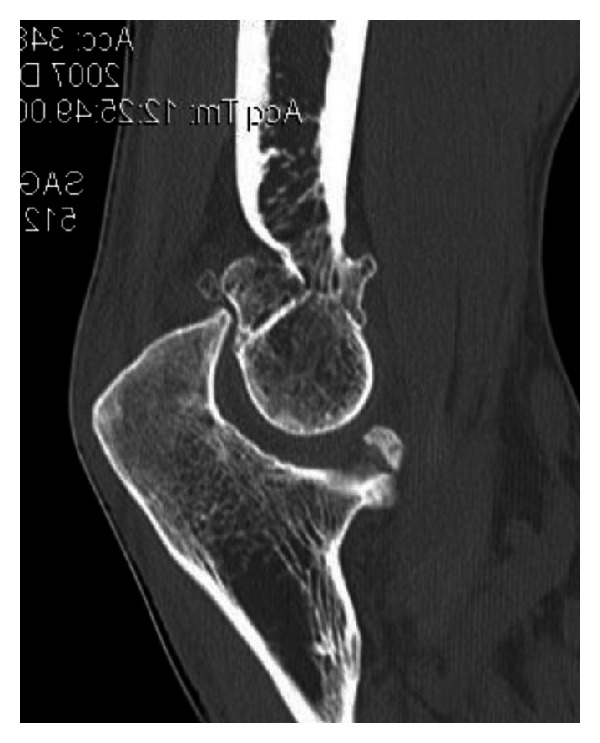
Sagittal CT image demonstrating osteophytes within the olecranon and coronoid fossae of the distal humerus.

Computerized tomography (CT) is frequently useful, especially when HO or intra-articular loose bodies are suspected, or if bony deformity or malunion is suspected in the setting of previous fracture. CT reconstruction is typically helpful in further delineating bony and articular anatomy (Figure 3). Advanced imaging is important in documenting ulnohumeral joint congruency as well as any osseous impingement secondary to overgrowth in the olecranon or coronoid fossae that would directly serve to limit motion. Magnetic resonance imaging (MRI) has a very limited role except to potentially workup subtle posttraumatic instability.

## 4. Nonoperative Treatment

Nonoperative management remains the mainstay of initial treatment for both primary osteoarthritis of the elbow and posttraumatic arthritis of the elbow and typically includes elbow sleeves, nonsteroidal anti-inflammatory medications, and intra-articular corticosteroid injections. For early-stage primary arthritis, symptoms are strongly associated with specific activities (weight-lifting, boxing, etc.), and counseling patients on avoidance of aggressive terminal flexion and extension exercises can result in substantial relief of pain. A course of supervised rehabilitation by a certified therapist is reserved typically for patients presenting with an acute-on-chronic presentation of symptoms with an associated effusion and limitations in motion.

Few reports have evaluated the role of hyaluronic acid as nonoperative management of elbow arthritis. A limited report on 18 patients demonstrated modest relief of pain at 3 months but no long-term benefit following the intraarticular injection of hyaluronic acid in the arthritic elbow [5].

## 5. Operative Treatment

Surgical management is indicated for patients with elbow pain or significant loss of mobility with resultant impairment of upper extremity function and limitation with daily activities. Careful consideration of patients' primary complaints is important in selecting the appropriate surgical intervention. Patients who continue to experience stiffness rather than pain despite nonoperative management may experience improvements in upper extremity function by surgical treatment, either open or arthroscopic debridement and capsular release. Similarly, patients with pain only the extremes of terminal flexion and/or extension but not in the midarc of elbow motion may benefit most from debridement as opposed to those with pain throughout the motion arc, which suggests arthritic involvement of the entire ulnohumeral joint. 

### 5.1. Surgical Debridement

From a purely mechanical standpoint, to improve elbow extension, posterior impingement must be removed between the olecranon tip and the olecranon fossa. Anteriorly, tethering soft tissues such as the anterior joint capsule and any adhesions between the brachialis and the humerus must be released. Similarly, to improve elbow flexion, the surgeon must release any posterior soft tissue structures that may be tethering the joint. They include the posterior joint capsule and the triceps muscle, which can become adherent to the humerus. The surgeon must remove any bony or soft-tissue impingement anteriorly, including any soft-tissue overgrowth in the coronoid and radial fossae. There must be a concavity above the humeral trochlea and capitellum to accept the coronoid centrally and the radial head laterally for full flexion to occur. These principles may be applied in the treatment of patients with the majority of their articular surfaces preserved but still complain of pain at the extremes of motion, as they will respond favorably to debridement of osteophytes anteriorly and/or posteriorly but with much of the articular surface well preserved.

Although a flexion contracture of at least 25 to 30 degrees and/or less than 110 to 115 degrees of active flexion was historically reported as an indication for surgical contracture release, surgical management may still be offered to certain patients who may desire full or nearly full motion for specific lifestyle and employment demands. Posttraumatic patients typically must be at least 3 to 4 months removed from injury to allow them to achieve “tissue equilibrium,” with maximal resolution of posttraumatic swelling and inflammation. Most importantly, patients must be willing to comply with the required extensive program of postoperative therapy, as operative outcomes depend on diligent participation in a structured rehabilitation program. 

Advances in elbow arthroscopy have resulted in favorable outcomes. Arthroscopic debridement of the elbow, particularly in a younger patient population, has excellent results with improvements in pain and range of motion [6–9] when the procedures are performed by surgeons with substantial experience with safe, meticulous techniques in elbow arthroscopy.

Open debridement should be considered in cases featuring severe elbow contractures with minimal joint motion, with ulnar nerve transposition surgery, and the presence of significant heterotopic bone. These patients are more reliably treated with extensive open surgery rather than arthroscopic debridement of the elbow to restore motion and protect the ulnar nerve. Open debridement, when required, is now commonly performed using medial and lateral exposures that provide adequate access for debridement and capsular release of the anterior and posterior elbow [10, 11]. 

Prior surgical exposure of the radial head might result in scarring and adhesions of the radial nerve to the anterior capsule, rendering the nerve susceptible to iatrogenic injury during arthroscopy. Open debridement is also indicated in this scenario. If the ulnohumeral joint shows marked degenerative changes, a simple release of the joint may not lead to improved motion and may exacerbate pain in an arthritic joint. If advanced posttraumatic arthritis is observed in the ulnohumeral articulation, salvage-type procedures are often required such as total elbow arthroplasty or soft-tissue interposition arthroplasty if surgery is undertaken [12].

Open ulnar nerve decompression with or without transposition should be considered along with open or arthroscopic elbow debridement if the patient has positive provocative neuropathic symptoms signs (i.e., a positive Tinel's test) or if the patient cannot flex past 100 to 110 degrees before surgery. Increased postoperative flexion following debridement theoretically should place the nerve at elevated risk of traction neuropathy [11].

Subtle elbow instability can commonly manifest as loss of motion after elbow fracture-dislocation; accordingly, special attention should be devoted towards evaluating elbow stability either with stability testing on physical examination or with stress radiographs. When instability is present, then treatment typically would include ligament reconstruction with or without capsular release if there is no arthritis present. In the setting of concomitant degenerative changes from long-standing instability, however, salvage arthroplasty options are considered. The stiff and unstable elbow is a particularly challenging condition to treat. 

### 5.2. Advanced Ulnohumeral Arthritis

Although the outcomes following arthroscopic and open debridement for elbow arthritis and stiffness are promising, these outcomes predominantly occurred in patients with moderate disease. Individuals with diffuse joint space narrowing and pain throughout the arc of motion are suggestive of more advanced arthrosis, which might not respond favorably to either arthroscopic or open elbow debridement. 

For patients with advanced disease who have failed nonoperative management, the primary surgical considerations include the age and demand level of the patient. Younger, high-demand patients with inflammatory arthropathy or severe posttraumatic or primary arthritis which affects the majority of the ulnohumeral articular surfaces are candidates for interposition arthroplasty or elbow arthrodesis. These procedures do not require postoperative lifting restrictions (2.3–4.5 kg) as total elbow arthroplasty and are best suited for a younger, high-demand cohort of patients. If the surgeon is considering interposition arthroplasty, elbow stability and adequate bone stock should be confirmed preoperatively, as this procedure may place the elbow at increased risk of postoperative instability [13].

**Figure 4:**
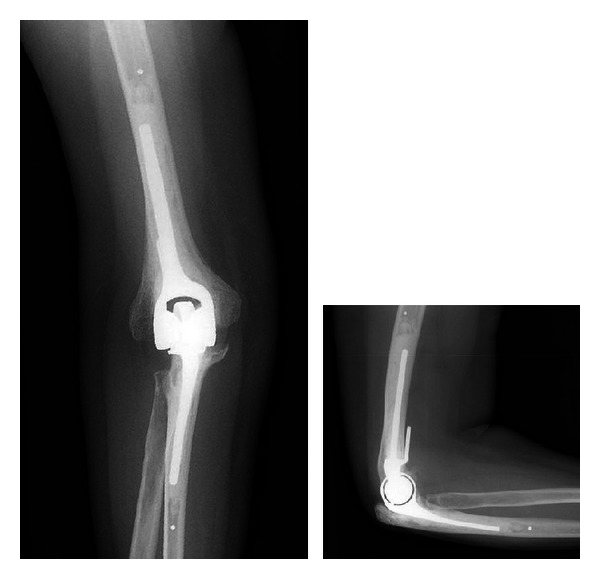
AP and lateral radiographs of a patient who underwent total elbow arthroplasty.

Total elbow arthroplasty is most appropriate for the low-demand, elderly patient (>60 year-old) with inflammatory, posttraumatic, or primary elbow arthritis. It is paramount that these patients understand and are compliant with the substantial postoperative lifting restrictions following total elbow arthroplasty (Figure 4) [14]. Total elbow arthroplasty is rarely applied in the setting of elbow osteoarthritis which occurs in a younger and typically male population. This procedure is most commonly applied for rheumatoid arthritis and comminuted distal humerus fractures in the elderly.

### 5.3. Radiocapitellar Arthritis

For the patients with symptomatic radiocapitellar arthritis who require surgical management, favorable outcomes following both open and arthroscopic radial head resection have been reported. Lateral elbow pain localized to the radiocapitellar joint with forearm rotation helps the practitioner identify patients with symptomatic arthritis of the radiocapitellar joint, and several authors have reported favorable outcomes of radial head resection [15, 16]. In asymptomatic patients who radiographically demonstrate radiocapitellar arthrosis but do not have lateral elbow pain localized to the radiocapitellar joint, the surgical results are less favorable as axial load across the joint is transferred to the ulnohumeral joint following radial head resection [9].

**Figure 5:**
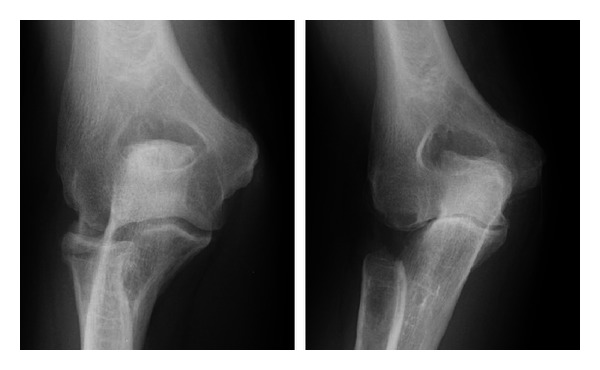
A young male laborer with symptomatic arthrosis of the radiocapitellar joint (A) underwent radial head resection; follow-up radiographs demonstrate progressive ulnohumeral arthritis approximately 18 months following the surgical procedure.

Although radial head resection does demonstrate favorable results in the appropriately selected patient, proximal migration of the radius may occur or progressive ulnohumeral arthritis may develop at the medial joint line owing to increased valgus forces at the elbow in younger higher demand patients (Figure 5). Radial head resection also predictably leads to decreased grip strength and can only be performed when there is no ligamentous elbow instability. While radial head replacement is widely utilized in the setting of comminuted radial head fracture, it is not effective in the setting of radiocapitellar arthritis since the capitellum has already degenerated. Radiocapitellar arthroplasty, including both radial head and capitellar replacement, is in the early stages of development to address this shortcoming but is not yet widely available. Innovations in implant design and surgical technique continue to evolve and outcomes are being more widely reported as surgical experience with these implants expands for use in treating radiocapitellar arthrosis [17, 18].

## 6. Summary

The evaluation of posttraumatic and primary osteoarthritis of the elbow requires a thorough, systematic approach to help accurately characterize the primary complaint and formulate a carefully considered treatment plan which accounts for the activity level and functional requirements of a given patient. Nonoperative treatment is almost always initiated although surgical treatment may be indicated in cases refractory to conservative management. Younger patients who experience pain or loss of motion in early stage ulnohumeral arthritis have experienced favorable outcomes following both arthroscopic and open elbow debridement although arthroscopic debridement should only be performed by surgeons who possess the advanced technical skills necessary for safe elbow arthroscopy. Patients with more advanced arthrosis of the elbow commonly require salvage reconstructive procedures. Although total elbow arthroplasty may be performed in the older, low-demand patient, the procedure may not be appropriate for younger, active patients who may not comply with substantial lifting restrictions required for longevity of the implants. For high-demand patients with advanced elbow arthritis, interposition arthroplasty or arthrodesis may provide acceptable functional outcomes while avoiding activity restrictions required by arthroplasty.
